# Mini G protein probes for active G protein–coupled receptors (GPCRs) in live cells

**DOI:** 10.1074/jbc.RA118.001975

**Published:** 2018-03-09

**Authors:** Qingwen Wan, Najeah Okashah, Asuka Inoue, Rony Nehmé, Byron Carpenter, Christopher G. Tate, Nevin A. Lambert

**Affiliations:** From the ‡ Department of Pharmacology and Toxicology, Medical College of Georgia, Augusta University, Augusta, Georgia 30912,; the § Graduate School of Pharmaceutical Sciences, Tohoku University, Sendai, Miyagi 980-8578 Japan, and; the ¶ MRC Laboratory of Molecular Biology, Cambridge CB20QH, United Kingdom

**Keywords:** G protein–coupled receptor (GPCR), G protein, arrestin, biosensor, molecular pharmacology, BRET, mini G protein, NanoLuc, protein complementation

## Abstract

G protein–coupled receptors (GPCRs) are key signaling proteins that regulate nearly every aspect of cell function. Studies of GPCRs have benefited greatly from the development of molecular tools to monitor receptor activation and downstream signaling. Here, we show that mini G proteins are robust probes that can be used in a variety of assay formats to report GPCR activity in living cells. Mini G (mG) proteins are engineered GTPase domains of Gα subunits that were developed for structural studies of active-state GPCRs. Confocal imaging revealed that mG proteins fused to fluorescent proteins were located diffusely in the cytoplasm and translocated to sites of receptor activation at the cell surface and at intracellular organelles. Bioluminescence resonance energy transfer (BRET) assays with mG proteins fused to either a fluorescent protein or luciferase reported agonist, superagonist, and inverse agonist activities. Variants of mG proteins (mGs, mGsi, mGsq, and mG12) corresponding to the four families of Gα subunits displayed appropriate coupling to their cognate GPCRs, allowing quantitative profiling of subtype-specific coupling to individual receptors. BRET between luciferase–mG fusion proteins and fluorescent markers indicated the presence of active GPCRs at the plasma membrane, Golgi apparatus, and endosomes. Complementation assays with fragments of NanoLuc luciferase fused to GPCRs and mG proteins reported constitutive receptor activity and agonist-induced activation with up to 20-fold increases in luminescence. We conclude that mG proteins are versatile tools for studying GPCR activation and coupling specificity in cells and should be useful for discovering and characterizing G protein subtype–biased ligands.

## Introduction

G protein–coupled receptors (GPCRs)^5^ signal by coupling to heterotrimeric G proteins and arrestins, which in turn activate or inhibit enzymes, kinases and other effector molecules to regulate nearly every aspect of cell function (1). The past 20 years have seen the development of a wide array of genetically-encoded optical sensors and probes to monitor nearly every step of these signaling cascades in living cells (2). An early example of this was the development of arrestin–fluorescent protein conjugates that translocate from the cytosol to the plasma membrane upon activation of cell-surface GPCRs (3). There are now many variants of this basic approach with different reporters and detection modalities, and these relatively simple tools have been used to gain tremendous insight into the functional properties of arrestins and GPCRs. Similar tools have been developed to monitor interactions between GPCRs and G proteins (4, 5), but compared with arrestins, the properties of heterotrimers are less favorable for many applications in cells. Heterotrimeric G proteins are membrane-associated (or tethered) proteins and therefore do not change subcellular location upon binding to GPCRs. This prevents simple visualization of complex formation in imaging experiments and produces background signals when receptor–G protein complexes are detected using resonance energy transfer (*e.g.* FRET and BRET) (4, 5). Moreover, receptor–G protein complexes in cells are short-lived. Ambient concentrations of guanine nucleotides lead to rapid complex dissociation, thus limiting signals generated by receptor–G protein association.

**Figure 1.**
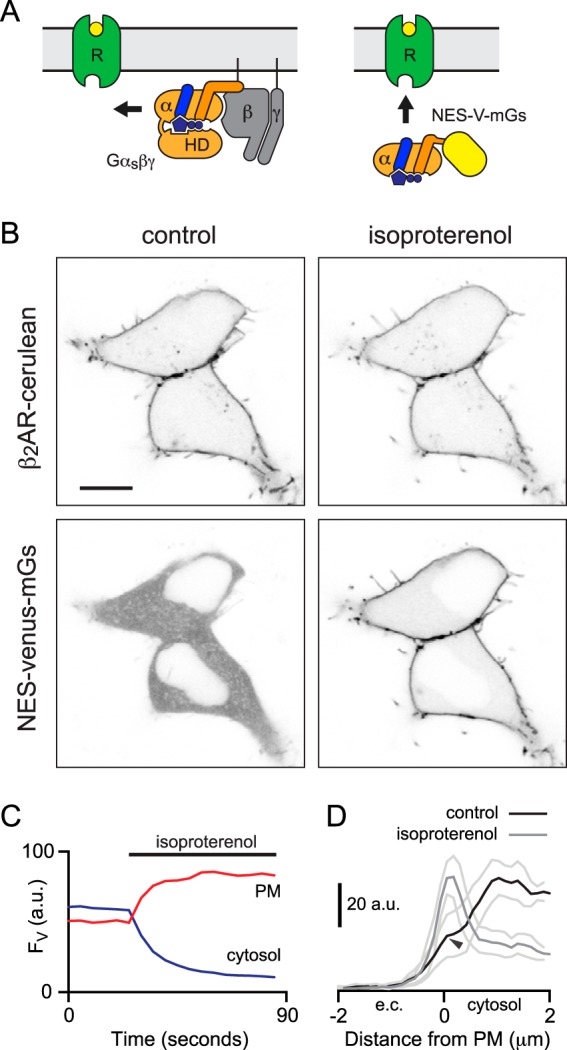
**Mini G proteins are recruited to active receptors at the plasma membrane.** *A,* cartoon representation highlighting the differences between G protein heterotrimers, which diffuse within the membrane to engage receptors (*left*), and mG proteins, which diffuse through the cytosol to engage receptors (*right*). Mini G proteins lack membrane anchors, N-terminal Gβγ-binding surface, and the α-helical domain (*HD*). *B,* confocal images of HEK 293 cells expressing cerulean-tagged β_2_-adrenergic receptors (β_2_AR-cerulean; *top panels*) and NES–venus–mGs (*bottom panels*). NES–venus–mGs is recruited to the plasma membrane after stimulation with 10 μm isoproterenol. *Scale bar,* 10 μm. *C,* venus fluorescence (*F*_V_, arbitrary units (*a.u.*)) at the plasma membrane (*PM*) and in the cytosol plotted against time for the cells shown in *B. D,* mean NES–venus–mGs fluorescence (± S.E.) line profiles drawn perpendicular to the plasma membrane from the extracellular (*e.c.*) space to the cytosol in five cells before and after application of isoproterenol. Weak accumulation of NES–venus–mGs at the plasma membrane is detectable prior to stimulation (*black arrowhead*).

Here, we report that mini G (mG) proteins (6, 7) are useful G protein surrogates for studies of GPCR activation in cells. Mini G proteins are Gα subunits with several key modifications as follows: 1) a truncated N terminus, which deletes membrane anchors and Gβγ-binding surface; 2) deletion of the α-helical domain; 3) mutations that improve protein stability *in vitro*; and 4) a mutation in the C-terminal α5 helix that uncouples GPCR binding from nucleotide release, thus stabilizing receptor–mG complexes in the presence of guanine nucleotides (Fig. 1*A*). Several mG variants also incorporate mutations at the GPCR–G protein interface and thus maintain the receptor-coupling specificity of the following four Gα subunit families: G_s_, G_i/o_, G_q/11_, and G_12/13_. These modifications enable mini G proteins to report receptor activation in living cells in much the same way as arrestins and conformation-specific nanobodies (8). Therefore, mG proteins will likely become broadly useful tools to study GPCR activation and receptor–G protein-coupling specificity in cells.

## Results and discussion

Mini G proteins were originally engineered for high-level expression in *Escherichia coli*, high stability *in vitro*, and effective coupling to GPCRs. To visualize mG protein expression in mammalian cells, we fused the fluorescent protein venus (9) to the N terminus of several mG proteins and expressed the resulting fusion proteins in HEK 293 cells under the control of a CMV promoter. Confocal microscopy revealed that mG variants shown previously to express well in *E. coli* (*e.g.* mGs_393) (6) were located diffusely throughout the cytoplasm and nucleus, whereas mG variants that express poorly in *E. coli* (*e.g.* mGq) (7) formed intracellular aggregates. Therefore, for this study we used four mG protein variants that express well in *E. coli* and HEK cells, specifically mGs_393, mGsi_43 (a chimera of mGs and mGi), mGsq_71 (a chimera of mGs and mGq), and mG12_8 (referred to hereafter as mGs, mGsi, mGsq, and mG12 for simplicity) (7). Because mG proteins in the nucleus are not immediately accessible to receptors at the plasma membrane, we added an N-terminal nuclear export sequence (NES) to second-generation mG fusion proteins, thereby restricting localization to the cytosol (Fig. 1*B*).

NES–venus–mG proteins bound tightly to agonist-activated GPCRs in intact cells. For example, stimulation of β_2_-adrenergic receptors fused to the fluorescent protein cerulean (β_2_AR–cerulean) with a saturating concentration of the agonist isoproterenol resulted in rapid translocation of NES–venus–mGs from the cytosol to the plasma membrane (Fig. 1, *B* and *C*). To estimate the stoichiometry of receptor–mG complexes at steady state, we used standardized confocal imaging conditions to measure cerulean and venus fluorescence at the plasma membrane of cells expressing cerulean-β_2_AR and venus–mGs in the presence of 10 μm isoproterenol. We calibrated these intensity measurements with a standard protein consisting of an extracellular cerulean, a transmembrane domain, and an intracellular venus (C-TM-V). The result indicated an average venus–mGs/cerulean–β_2_AR stoichiometry of ∼1:1 (mean venus/cerulean = 1.17; 95% confidence interval 0.87–1.47; *n* = 17). This is likely to be an overestimate due to the presence of residual cytosolic venus–mGs near the plasma membrane. Nevertheless, recruitment of mGs to active β_2_ARs was clearly efficient, consistent with the formation of relatively stable receptor–mG complexes. In some cells, there was detectable accumulation of mG proteins at the plasma membrane prior to stimulation (Fig. 1*D*), suggesting that mG proteins could also bind to ligand-free GPCRs (see below).

### BRET between GPCRs and mG proteins

**Figure 2.**
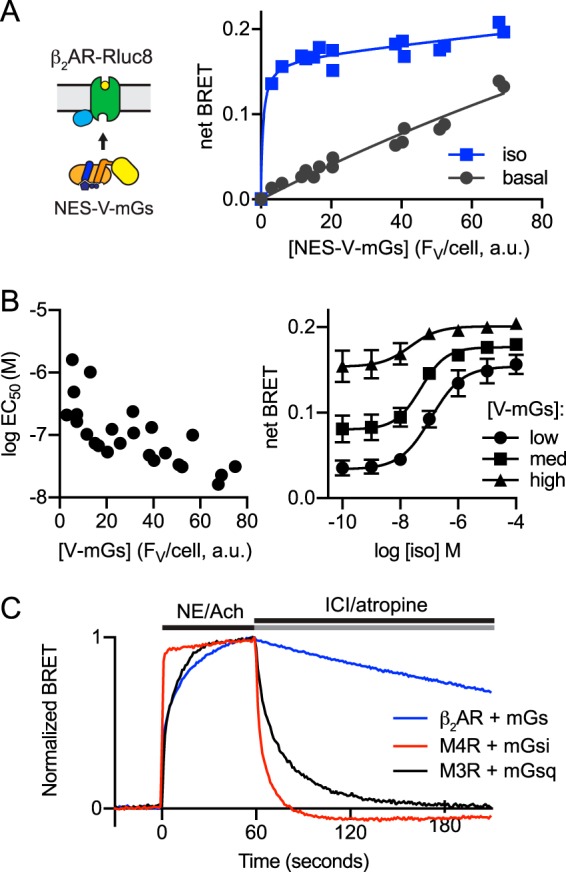
**BRET between β_2_AR–Rluc8 and NES–venus–mGs.** *A,* net BRET is plotted *versus* mean NES–venus–mGs fluorescence intensity per cell (*F*_V_, arbitrary units (*a.u.*)) for control cells and cells stimulated with 10 μm isoproterenol (*iso*). Cells were transfected with a constant amount of DNA encoding β_2_AR–Rluc8, and an increasing amount of DNA encoding NES–venus–mGs. Data are fitted to a two-site–specific binding equation, and data points from three independent experiments are superimposed. *B,* log EC_50_ is plotted *versus* mean NES–venus–mGs fluorescence intensity per cell (*F*_V_, arbitrary units) for five independent experiments with five different expression levels each (*left*). Example concentration-response curves are shown for cells expressing low (EC_50_ = 116 nm), medium (EC_50_ = 52 nm), and high (EC_50_ = 22 nm) levels of NES–venus–mGs (*right*); mean ± S.E. of three independent experiments. *C,* normalized BRET is plotted *versus* time for cells expressing β_2_AR-, M4R-, and M3R-Rluc8 together with NES–venus–mGs, –mGsi, and –mGsq; acetylcholine (100 μm; *Ach*), (−)-norepinephrine (10 μm), ICI 118,551 (10 μm), and atropine (10 μm) were added as indicated. Traces are the average of 4–7 experiments.

BRET is widely used to monitor protein interactions in cells (10) and can also detect protein trafficking or translocation to membrane compartments (11). To demonstrate the utility of mG proteins for BRET experiments, we cotransfected cells with a fixed amount of plasmid DNA encoding β_2_-adrenergic receptors fused to the *Renilla* luciferase Rluc8 (β_2_AR–Rluc8) and increasing amounts of DNA encoding NES–venus–mGs, and we quantified expression of the latter by flow cytometry. In unstimulated cells, there was a shallow monotonic increase in BRET as NES–venus–mGs expression increased, consistent with low- affinity binding of mGs to either β_2_ARs or the plasma membrane. Stimulation with a saturating concentration of isoproterenol led to a pronounced increase in energy transfer that was proportionally greater when NES–venus–mGs expression was lower (Fig. 2*A*). BRET in unstimulated cells approaches the BRET observed in agonist-stimulated cells when NES–venus–mGs expression is high. This suggests that the former signal is largely due to specific binding to β_2_AR–Rluc8 rather than nonspecific membrane binding, as specific and nonspecific signals would be expected to be additive. In the presence of isoproterenol, the BRET signals were nearly maximal when NES–venus–mGs expression was low, again consistent with stable, high-affinity β_2_AR–mGs complexes under these conditions. However, a shallow increase in BRET efficiency in the presence of agonist could still be discerned as NES–venus–mGs expression increased, consistent with a superimposed (and much smaller) low-affinity binding component (Fig. 2*A*). The origin of this second component is uncertain, but we speculate that it represents low-affinity binding of NES–venus–mGs to β_2_AR–Rluc8 receptors that are located in intracellular compartments (*e.g.* the Golgi apparatus) and therefore do not have access to extracellular isoproterenol.

Mini G proteins functionally mimic the nucleotide-empty, GPCR-bound state of G protein heterotrimers (6), and therefore, mG and agonist binding to GPCRs should be mutually cooperative. Consistent with this expectation, we found that increasing NES–venus–mGs expression led to a 5–10-fold leftward shift (decreased EC_50_) in isoproterenol *versus* BRET concentration-response curves (Fig. 2*B*). As noted above, increasing NES–venus–mGs expression also increased basal BRET signals and decreased the assay's dynamic range. Therefore, interactions between mG proteins and GPCRs are sensitive to mG protein abundance in a manner that is consistent with the allosteric model of GPCR–G protein-coupling (12).

We next tested the reversibility of agonist-induced recruitment of mG proteins by measuring BRET during sequential application of an agonist and an antagonist or inverse agonist. We found that β_2_AR–Rluc8/NES–venus–mGs complexes were quite stable in the presence of norepinephrine and required more than 15 min to dissociate after addition of the inverse agonist ICI 118,551 (Fig. 2*C*). This is much slower than dissociation of receptor–heterotrimer complexes in intact cells (2), and it likely reflects stabilization of the orthosteric ligand-binding site by the presence of a surrogate (mG protein) that mimics a nucleotide-empty G protein (13). In contrast, muscarinic acetylcholine M3R–Rluc8/NES–venus–mGsq and M4R–Rluc8/NES–venus–mGsi complexes dissociated much more rapidly after addition of atropine (Fig. 2*C*), indicating that the stability of agonist–receptor–mG complexes is widely variable.

**Figure 3.**
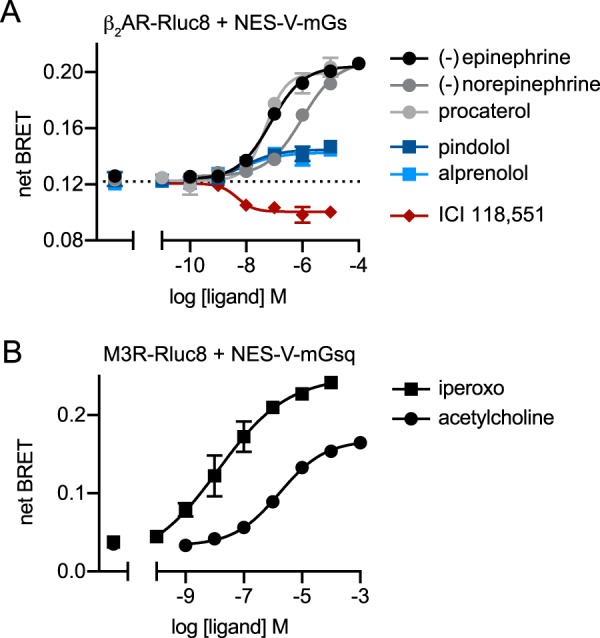
**BRET between GPCRs and mG proteins reports the full range of ligand efficacy.** Net BRET between β_2_AR–Rluc8 and NES–venus–mGs (*A*) and M3R–Rluc8 and NES–venus–mGsq (*B*) is plotted *versus* log concentration for the indicated ligands and fitted to a four-parameter logistic equation; mean ± S.E. of four independent experiments. Data points at the *far left* of these panels represent vehicle controls.

One practical advantage of assays that directly monitor GPCR–transducer coupling as opposed to downstream signals is their ability to report both ligand potency and efficacy without the potential confounds of spare receptors or assay readouts that are not linearly related to efficacy. We found that BRET between β_2_AR–Rluc8 and NES–venus–mGs accurately reported the difference in potency of the full agonists (−)-epinephrine and (−)-norepinephrine at this receptor, as well as weak partial agonist activity of ligands that are typically classified as partial agonists (pindolol) or antagonists (alprenolol) (14). Partial agonist activity of alprenolol is somewhat surprising but consistent with a previous report that binding of this ligand to β_2_AR is promoted by nanobody 80 (Nb80), a G protein surrogate that stabilizes the active state of the receptor (15). Notably, the inverse agonist ICI 118,551 decreased BRET between β_2_AR–Rluc8 and NES–venus–mGs (Fig. 3*A*), again supporting the notion that in unstimulated cells this signal at least partly reflects specific binding of NES–venus–mGs to ligand-free β_2_AR–Rluc8. We also found that iperoxo induced a greater maximum BRET signal between M3R–Rluc8 and NES–venus–mGsq than the native ligand acetylcholine (Fig. 3*B*). This suggests that the former ligand acts as a superagonist at this receptor, similar to what has been reported for the M2 acetylcholine receptor (16). Taken together, these results suggest that mG proteins report the full range of ligand efficacy at GPCRs.

### Mini G protein subtypes maintain appropriate coupling specificity

**Figure 4.**
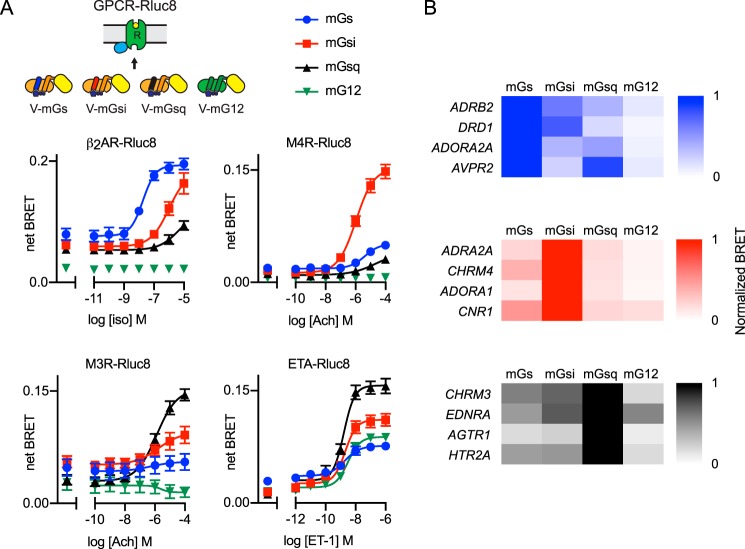
**Mini G protein subtypes maintain appropriate coupling specificity.** *A,* net BRET to four different NES–venus–mG subtypes is plotted *versus* log ligand concentration for β_2_-adrenergic receptors (β*_2_AR–Rluc8*), M4 and M3 acetylcholine receptors (*M4R-Rluc8* and *M3R-Rluc8*), and endothelin A receptors (*ETA-Rluc8*). Ligands are isoproterenol (*iso*), acetylcholine (*Ach*), and endothelin-1 (*ET-1*); mean ± S.E. of 3–4 independent experiments. Data points at the *far left* of each panel represent vehicle controls. *B,* heat maps representing normalized maximal BRET (which includes both constitutive and agonist-induced signals, normalized to the best-responding mG protein) for 12 receptors (fused to Rluc8) paired with NES–venus–mG proteins. Heat maps for canonical G_s_–, G_i/o_–, and G_q_–coupled receptors are shown in *blue, red,* and *black*, respectively; *n* = 3–5 independent experiments for each receptor.

Because there are four families of Gα subunits, it was necessary to develop mG proteins that could be surrogates for heterotrimers from each family. The mutations incorporated into mGs were transferable to some Gα subunits (*e.g.* G_12_) but not others (*e.g.* G_q_ and G_i1_) (7), and therefore mGsq and mGsi chimeras were developed wherein specificity-determining residues in mGs were replaced with residues corresponding to G_q_ and G_i1_. *In vitro,* these chimeras gain coupling to G_q_–coupled and G_i_–coupled receptors, respectively, and lose coupling to G_s_–coupled receptors (7). To demonstrate the ability of mG protein subtypes to couple to appropriate GPCRs in cell-based assays, we profiled BRET between four NES–venus–mG variants (mGs, mGsi, mGsq, and mG12) expressed at similar levels (53 ± 21, 59 ± 15, 36 ± 14, and 42 ± 16 arbitrary fluorescence units, respectively; mean ± S.D., *n* = 4) and four receptors that collectively couple to all four G protein subtypes. We found that mG proteins maintained appropriate coupling specificity as defined by the primary transducer annotation in the BPS/IUPHAR Guide to Pharmacology (Fig. 4*A*) (14). Importantly, mG proteins reported not only known primary coupling interactions (*e.g.* β_2_AR and mGs) but also known secondary coupling interactions with either lower potency or maximal response (*e.g.* β_2_AR and mGsi). We have observed appropriate subtype-specific coupling of mG proteins to several additional receptors (Fig. 4*B*), and we have not observed examples of clearly inappropriate coupling, suggesting that the changes made to mGsi and mGsq included key specificity determinants. These results suggest that mG proteins will be useful for quantifying the efficiency of coupling between GPCRs and G protein subtypes.

**Figure 5.**
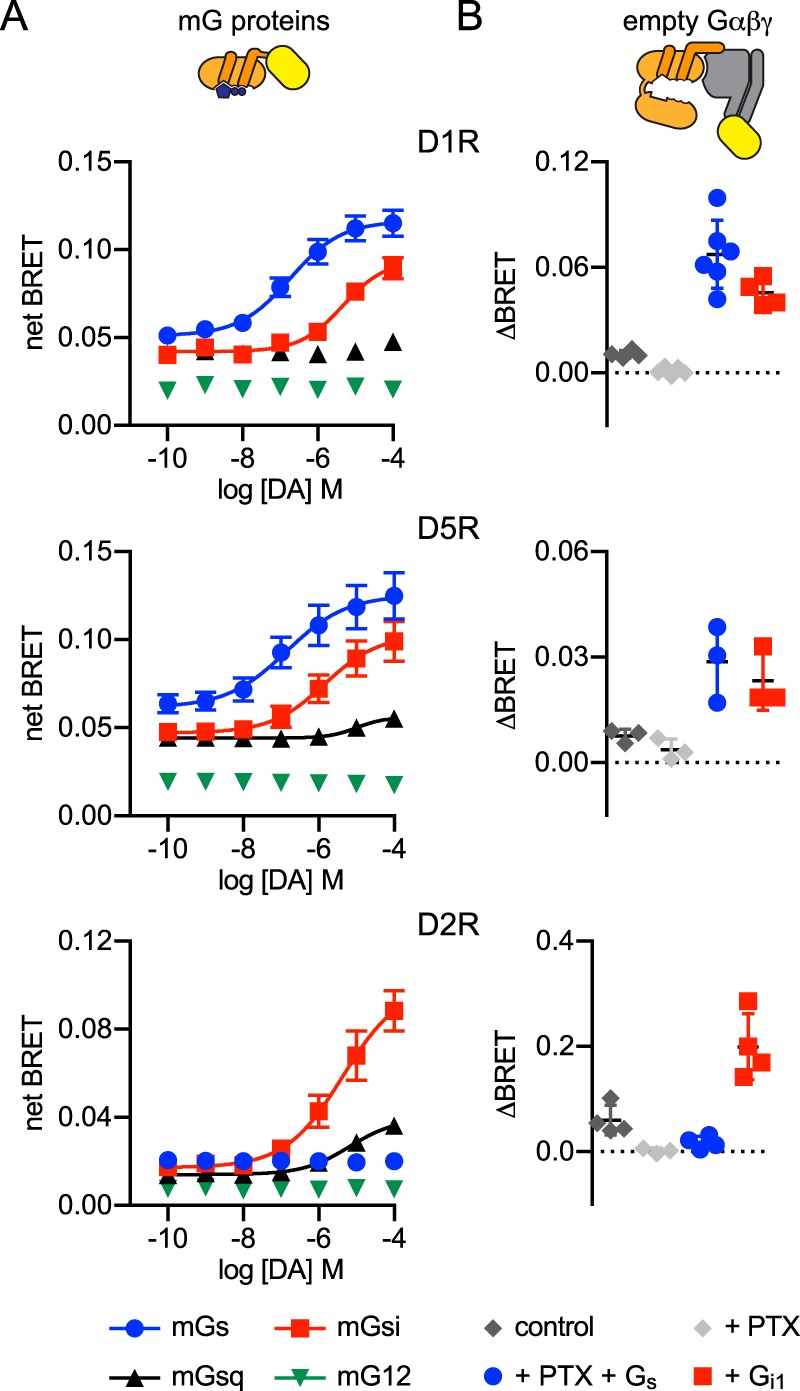
**Secondary coupling of G_s_–coupled dopamine receptors to mGsi and G_i1_ heterotrimers.** *A,* recruitment of mG proteins to dopamine receptors. Net BRET between D1R–, D5R–, or D2R–Nluc and four different NES–venus–mG subtypes in response to dopamine (*DA*) is shown; mean ± S.E. of 5–7 independent experiments. *B,* recruitment of empty heterotrimers to dopamine receptors. The difference (Δ*BRET*) between net BRET observed in the presence of 0.5 mm GDP alone and in the presence of apyrase and dopamine (100 μm) is shown. Cells lacking endogenous Gα_s_, Gα_q_, and Gα_12_ subunits expressed D1R-, D5R-, or D2R-Rluc8 and heterotrimers consisting of Gβγ–venus and the remaining endogenous Gα subunits (*control*) or overexpressed Gα_s_ or Gα_i1_. In some experiments cells also expressed the S1 subunit of pertussis toxin (*PTX*); mean ± S.D. of 3–6 independent experiments.

Indeed, when we extended this analysis to other receptors, we noticed secondary coupling interactions that were previously unknown or unappreciated. For example, we found that both D1 and D5 dopamine receptors (D1R and D5R) coupled primarily to mGs but also secondarily to mGsi (Fig. 5*A*). Other closely-related G_s_–coupled monoamine receptors (β_2_AR and β_1_AR) are known to couple to both G_s_ and G_i/o_ heterotrimers, but we found only one previous report of D1R coupling to G_i_ (17) and no reports of D5R coupling to G_i_. To verify D1R and D5R coupling to G_i_ proteins, we measured BRET between luciferase-tagged receptors and “empty” (nucleotide-free) heterotrimers consisting of Gβγ-venus and either Gα_s_ or Gα_i1_. To minimize contributions from endogenous Gα subunits, we used HEK cells with CRISPR/Cas9-mediated deletion of Gα_s_, Gα_q/11_, and Gα_12/13_ subunits (18). Minimal BRET signals were generated when only the remaining endogenous Gα subunits were available to form heterotrimers with overexpressed Gβγ-venus (*control* in Fig. 5*B*), either with or without coexpression of pertussis toxin S1 subunit to uncouple endogenous Gα_i/o_ subunits. In contrast, substantial agonist-dependent BRET signals were observed when either Gα_s_ or Gα_i1_ were coexpressed with Gβγ–venus (Fig. 5*B*). For comparison, we also studied coupling of mG proteins and empty heterotrimers to D2 dopamine receptors, and we found that these receptors, as expected, coupled to both mGsi and G_i1_ heterotrimers but not mGs or G_s_ heterotrimers (Fig. 5*A*). Although caution is warranted with respect to drawing conclusions based solely on mG proteins, our results suggest that these tools will be useful for studying G protein–coupling specificity. Our results also suggest that G_s_–coupled adrenergic and dopamine receptors share the property of dual coupling to G_s_ and G_i/o_ heterotrimers, and therefore the possibility that G_i/o_ heterotrimers mediate some of the actions of D1R and D5R warrants further study.

### Mini G proteins bind to active GPCRs in intracellular compartments

**Figure 6.**
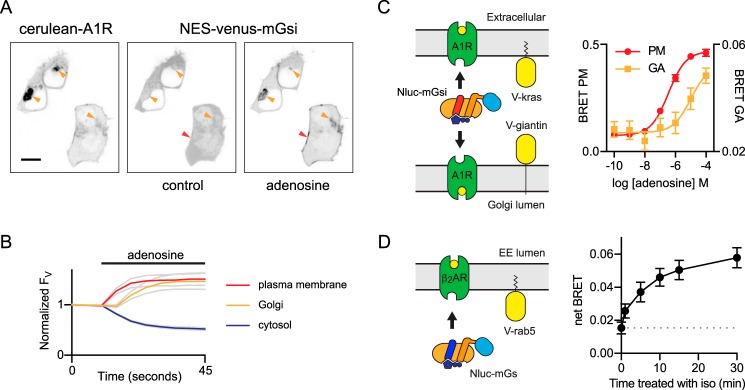
**Mini G proteins are recruited to active receptors at the Golgi apparatus.** *A,* confocal images of cells expressing cerulean-tagged A_1_-adenosine receptors (*left*) and NES–venus–mGsi before (*center*) and after (*right*) stimulation with 100 μm adenosine. Some cells retain significant cerulean–A1R in the Golgi apparatus, and stimulation with adenosine recruits NES–venus–mGsi to this compartment (*orange arrowheads*) as well as to the plasma membrane (*red arrowhead*). *Scale bar,* 10 μm. *B,* mean NES–venus–mGsi fluorescence (*F*_V_, arbitrary units; ± S.E.) at the plasma membrane, Golgi apparatus, and in the cytosol plotted against time for nine cells similar to those shown in *A*. Accumulation of NES–venus–mGsi at the Golgi apparatus was delayed ∼5 s compared with the plasma membrane. *C,* BRET between NES–NanoLuc–mGsi and either venus-kras (*V-kras*) at the plasma membrane or venus-giantin (*V-giantin*) at the Golgi apparatus (*GA*) in response to stimulation of unlabeled A1Rs; mean ± S.D. of three independent experiments. *D,* BRET between NES-Nluc-mGs and the early endosome marker venus-rab5 is plotted *versus* time after stimulation of unlabeled β_2_AR with 10 μm isoproterenol; mean ± S.E. of four independent experiments.

Nanobodies raised against active-state GPCRs have been used to detect active receptors in cells, including receptors located in intracellular compartments (8, 19). Not surprisingly, we found that mG proteins could be used in a similar manner to indicate GPCR activation in intracellular compartments. For example, CMV promoter-driven overexpression of cerulean-tagged A_1_-adenosine receptors (cerulean–A1R) often led to accumulation of this receptor in perinuclear compartments that we presumptively identified as the Golgi apparatus. Stimulation with adenosine recruited NES–venus–mGsi not only to the plasma membrane but also to the Golgi apparatus (Fig. 6*A*). The onset of NES–venus–mGsi recruitment to the Golgi was delayed by ∼5 s compared with the onset of NES–venus–mGsi recruitment to the plasma membrane (Fig. 6*B*). To confirm the presence of active A1Rs in the Golgi apparatus, we measured BRET between the luciferase NanoLuc fused to mGsi (NES–Nluc–mGsi) and venus-tagged acceptor molecules directed specifically to either the plasma membrane (venus–kras) or the Golgi apparatus (venus–giantin) (11) in cells expressing unlabeled A1Rs (Fig. 6*C*). Adenosine produced a concentration-dependent increase in BRET to both membrane markers, although this effect was less potent for recruitment to the Golgi apparatus (Fig. 6*C*). Taken together, these results suggest that extracellular adenosine has rapid access to the Golgi lumen in HEK 293 cells and that overexpressed A1Rs are functional in this compartment. The former observation is somewhat surprising in light of the hydrophilic character of adenosine (calculated octanol-water partition coefficient = −1.1) and suggests that HEK 293 cells express nucleoside transporters capable of delivering adenosine to the Golgi lumen (19, 20). Using a similar approach, we could also follow the accumulation of active β_2_ARs in early endosomes after prolonged stimulation with agonist (Fig. 6*D*) (8).

### Mini G proteins support luciferase complementation

**Figure 7.**
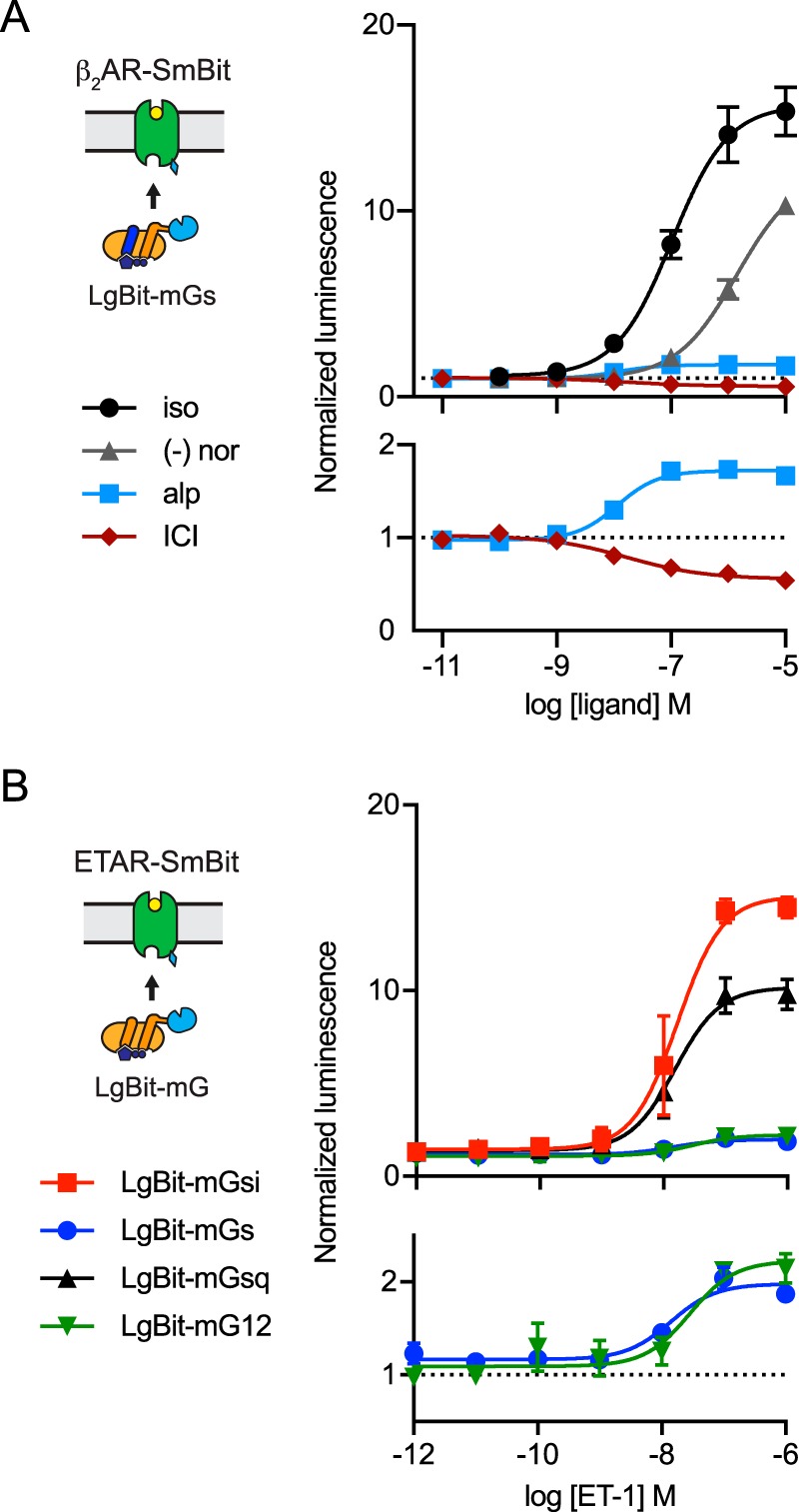
**Split luciferase complementation driven by mGs.** *A,* total luminescence (normalized) emitted by cells cotransfected with β_2_AR-SmBit and LgBit-mGs is plotted *versus* ligand concentration for isoproterenol (*iso*), (−)-norepinephrine (*nor*), alprenolol (*alp*), and ICI 118,551 (*ICI*); mean ± S.E. of 4–13 independent experiments. *B,* total luminescence (normalized) emitted by cells cotransfected with ETAR–SmBit and four different LgBit–mG proteins is plotted *versus* the concentration of endothelin-1 (*ET-1*); mean ± S.D. of four independent experiments.

For many screening applications, split luciferase reporters are preferred because of their single wavelength output, high signal/background ratio, and high sensitivity. One recently-developed technology is complementation of 11-amino acid (SmBit) and 18-kDa (LgBit) fragments of the engineered luciferase NanoLuc (Nluc) (21). The affinity of SmBit for LgBit is low enough that efficient complementation in cells requires fusion of these fragments to interacting proteins. We explored the possibility of using this system to report the agonist-dependent interaction of mG proteins and GPCRs by fusing LgBit and SmBit fragments to the N terminus of mG proteins and the C terminus of β_2_AR. Between the two possible orientations, we found that the most efficient complementation took place when LgBit was fused to mG proteins (*e.g.* LgBit-mGs), and SmBit was fused to the receptor (β_2_AR–SmBit). In individual experiments, agonist stimulation produced as much as a 20-fold increase in luminescence intensity. As was the case with BRET assays, NanoLuc complementation faithfully reported full agonist, partial agonist, and inverse agonist activity at β_2_AR-SmBit (Fig. 7*A*). This strategy was also applicable to LgBit-mGsi, -mGsq, and -mG12, as all of these fusion proteins supported NanoLuc complementation with the promiscuous endothelin-A receptor, ETAR-SmBit (Fig. 7*B*). These results suggest that mG proteins should serve as useful vehicles for protein complementation assays.

In summary, although mG proteins originated as G protein surrogates for X-ray crystallography, their unique features present many opportunities for applications other than structural biology. In cellular assays, mG proteins should be useful for studying the determinants of receptor–G protein-coupling specificity, biased ligands, and activation of receptors at different subcellular locations. We expect that many additional uses will be found for mG proteins in cells and cell extracts and that mG proteins will prove to be valuable tools for diverse studies of GPCR biology.

## Experimental procedures

### Plasmid DNA constructs

Human codon-optimized fragments encoding mG sequences (7) were synthesized as gBlocks (Integrated DNA Technologies, Coralville, IA), amplified by PCR, and subcloned into the vector pVenus-C1 using BglII and EcoRI to produce venus–mG constructs. A nuclear export sequence and linker (underlined) (MLQNELALKLAGLDINKTGGSG) was later added to the N terminus of venus by QuikChange (Agilent Technologies, Santa Clara, CA) PCR insertional mutagenesis to produce NES–venus–mG constructs. A similar strategy was used to produce NES–Nluc–mG plasmids using the vector pNluc–C1. LgBit–mG constructs were made by amplifying mG fragments by PCR and subcloning into the vector pBiT1.1-N (Promega Corp., Madison, WI) with XhoI and EcoRI. β_2_AR–SmBit was made by adding a linker and SmBit (GGSGVTGYRLFEEIL) to the C terminus of the β_2_AR using QuikChange PCR. Additional GPCR–SmBit constructs were derived from β_2_AR–SmBit using a BamHI site incorporated into the GGSG linker. Plasmids encoding Gα subunits were obtained from cdna.org (Bloomsburg University, Bloomsburg, PA). A plasmid encoding the S1 subunit of pertussis toxin was kindly provided by Stephen R. Ikeda (NIAAA, National Institutes of Health, Rockville, MD). Plasmids encoding cerulean–TM–venus, venus–kras, venus–rab5, venus–giantin, and Gβγ–venus have been described previously and were used in this study under conditions that are essentially the same as described previously (11, 22–25). Several different GPCR–luciferase constructs were made by appending either Rluc8 or Nluc directly to the receptor C terminus either by QuikChange PCR or by subcloning into pRluc8-N1 or pNluc-N1 vectors. GPCR sequences were obtained either from cdna.org, as a gift from Jonathan Javitch (Columbia University, New York) or as a gift from Bryan Roth (26) (Addgene, PRESTO-Tango kit 1000000068). All plasmid constructs were verified by Sanger sequencing.

### Cell culture and transfection

HEK 293 cells (ATCC) were propagated in plastic flasks on 6-well plates and on polylysine-coated glass coverslips according to the supplier's protocol. HEK 293 cells with targeted deletion of *GNAS*, *GNAL*, *GNAQ*, *GNA11*, *GNA12*, and *GNA13* (ΔGs/ΔGq/ΔG12 cells) were derived and authenticated as described previously (18). Cells were transiently transfected in growth medium using linear polyethyleneimine (*M*_r_ 25,000; Polysciences Inc., Warrington, PA) at an nitrogen/phosphate ratio of 20 and were used for experiments 12–48 h later. Up to 3 μg of plasmid DNA was transfected in each well of a 6-well plate.

### Confocal imaging

Cells grown on 25-mm round coverslips were transferred to an imaging chamber and washed with DPBS. Drug solutions were added directly to the chamber by pipetting. Confocal images were acquired using a Leica (Wetzlar, Germany) SP8 scanning confocal microscope and a ×63, 1.4 NA objective. Venus was excited with a 488-nm diode laser and detected at 500–650 nm. Cerulean was excited with a 448-nm diode laser and detected at 460–520 nm.

### BRET, luminescence, and fluorescence measurements

Cells were washed with DPBS, harvested by trituration, and transferred to opaque black or white 96-well plates containing diluted drug solutions. For assays with nucleotide-free heterotrimers (Fig. 4), cells were washed with permeabilization buffer containing 140 mm KCl, 10 mm NaCl, 1 mm MgCl_2_, 0.1 mm K-EGTA, 20 mm NaHEPES (pH 7.2), harvested by trituration, and permeabilized in the same buffer containing 10 μg ml^−1^ high purity digitonin (EMD Millipore, Burlington, MA). Measurements were made from permeabilized cells supplemented either with GDP (0.5 mm) or apyrase (2 units ml^−1^; Sigma) and agonist. BRET and luminescence measurements were made using a Mithras LB940 photon-counting plate reader (Berthold Technologies GmbH, Bad Wildbad, Germany). Coelenterazine h (5 μm; Nanolight, Pinetop, AZ) or furimazine (Nano-Glo; 1:1000, Promega Corp.) were added to all wells immediately prior to making measurements with Rluc8 and Nluc (or Nluc fragments), respectively. Raw BRET signals were calculated as the emission intensity at 520–545 nm divided by the emission intensity at 475–495 nm. Net BRET was this ratio minus the same ratio measured from cells expressing only the BRET donor. NES–venus–mG fluorescence in Fig. 2 was measured using a Guava 6HT/2L flow cytometer (excitation 488 nm, detection 525/30 nm) and reported as average fluorescence from all positive cells.

## Author contributions

Q. W., N. O., and N. A. L. investigation; Q. W., N. O., A. I., R. N., B. C., and C. G. T. writing-review and editing; A. I., R. N., B. C., and C. G. T. resources; A. I., C. G. T., and N. A. L. funding acquisition; N. A. L. supervision; N. A. L. writing-original draft; N. A. L. project administration.

## Footnotes

5 The abbreviations used are:

GPCR: G protein–coupled receptor
BRET: bioluminescence resonance energy transfer
mG: mini G
NES: nuclear export sequence
DPBS: Dulbecco's phosphate-buffered saline
Nluc: NanoLuc
β_2_AR: β_2_-adrenergic receptor
D1R: D1 dopamine receptor
D5R: D5 dopamine receptor
A1R: A_1_-adenosine receptor.
